# Effect of Exercise-Related Factors on the Perception of Time

**DOI:** 10.3389/fphys.2020.00770

**Published:** 2020-07-06

**Authors:** David G. Behm, Tori B. Carter

**Affiliations:** School of Human Kinetics and Recreation, Memorial University of Newfoundland, St. John’s, NL, Canada

**Keywords:** temporal, work, time dilation, time constriction, arousal

## Abstract

The concept of time whether considered through the lenses of physics or physiology is a relative measure. Alterations in time perception can have serious implications in sport, fitness and work. Accurate perception of time is an important skill with many time constrained sports (i.e., basketball, North American football, tennis, gymnastics, figure skating, ice hockey, and others), and work environments (i.e., workers who need to synchronize their actions such as police and military). In addition, time distortions may play a role in exercise adherence. Individuals may be disinclined to continue with healthy, exercise activities that seem protracted (time dilation). Two predominant theories (scalar expectancy theory and striatal beat frequency model) emphasize the perception of the number of events in a period and the role of neurotransmitters in activating and coordinating cortical structures, respectively. A number of factors including age, sex, body temperature, state of health and fitness, mental concentration and exercise intensity level have been examined for their effect on time perception. However, with the importance of time perception for work, sport and exercise, there is limited research on this area. Since work, sports, and exercise can involve an integration of many of these aforementioned factors, they are interventions that need further investigations. The multiplicity of variables involved with work, sport, and exercise offer an underdeveloped but fruitful field for future research. Thus, the objective of this review was to examine physiological and psychological factors affecting human perception of time and the mechanisms underlying time perception and distortion with activity.

## Key Points

– Two major theories underlying the perception of time are the scalar expectancy theory and striatal beat frequency model, which emphasize the perception of the number of events in a period and the role of neurotransmitters in activating and coordinating cortical structures, respectively.
– The perception of time is relative and can be affected by age, sex, body temperature, state of health and fitness, mental concentration, and exercise intensity level.
– Physiological and psychological arousal, which are intrinsically linked with exercise and work also affect time perception. However, there is very little research examining the effect of the many types of exercise and activity on human time perception and thus this review is a call to further action in this area.

## Introduction

The concept of time has fascinated people for centuries, with ancient to modern philosophers and scientists unable to fully agree upon the definition and characteristics of the concept (Bunnag, 2017). With the development of atomic clocks with nanosecond precision, time is perceived by many to be a precise, objective measure. However, according to Albert Einstein’s theory of special relativity, time is relative (Einstein, 1905). Stephen Hawking’s work revealed that time can be influenced by gravitation and postulated that it may even cease to exist within the confines of a black hole or at or prior to the Big Bang (beginning of the universe) (Hawking and Hartle, 1972). To further cloud the issue, physicists might argue that without the existence of time, one cannot speak of a period prior to the Big Bang since time did not exist. Hence, although time within a specified space-time continuum can be precisely monitored, the existence or perception of time can fluctuate.

Recently, research has begun to focus on the human personal experience of time, known as time perception (Wittmann and Paulus, 2009). Human perception of time is an important dimension where people make decisions for everyday behavior and survival (Wittmann and Paulus, 2008). Time perception relates to our awareness of the passage of time; this experience is intertwined with environmental, psychological, and physiological processes (Wittmann and van Wassenhove, 2009; Wittman, 2013; Allman et al., 2014). For instance, it is a common perception that time passes more slowly when a person is bored or passes by more rapidly for adults than children (Burdick, 2017). While the neural basis for time perception is still unknown (Wittmann and van Wassenhove, 2009; Wittman, 2013), there are two predominant models used to describe the process of time perception; scalar expectancy theory, often called the pacemaker accumulator model, and the striatal beat frequency model (Allman and Meck, 2012; Allman et al., 2014). These models are used to highlight the effects of arousal (physiological or psychological) on the distortion of time (Lambourne, 2012; Jakubowski et al., 2015; Droit-Volet and Berthon, 2017).

Exercise is a form of physiological arousal (Duncan et al., 2016), however, there is limited research on the effects of exercise-induced arousal on time perception. Furthermore, there is no consensus on dual task exercise activities and time perception. For example, a study on obese children found that dual task conditions (listening to music while running and running in silence) slowed the internal clock and allowed them to run longer (De Bourdeaudhuij et al., 2002), whereas swimmers showed no effect on time perception during dual task activities (Tobin and Grondin, 2012). Additionally, discrepancies have been found in studies monitoring varied temperature effects on time perception. Some studies state that an increase in temperature causes the compression of time while others argue that this effect only occurs once a level of perceived fatigue is achieved (Tamm et al., 2014, 2015). Currently, there are no investigations on the effects of isometric, concentric or eccentric contractions, nor maximal exercise on time perception. It has also not been discerned whether there is a certain duration or intensity at which time perception changes or if there is an optimal level to exercise at, with reference to timing and time perception.

Additionally, exercise may interact with several other factors in the perceived distortion of time, including age, sex, body temperature, state of health and fitness, mental concentration and intensity level. Age differences have yet to be explored pertaining to time perception in exercise. Similarly, fitness level has not been researched yet as a factor that may attribute to improved or impaired time perception during exercise. Even so, task knowledge has been linked to improved time perception and a higher task knowledge is often possessed by highly trained individuals (Tobin and Grondin, 2012). In relation to sex differences, the limited research suggests women may perceive time to pass more slowly than men during exercise (Pleil et al., 2011; Hanson and Buckworth, 2016), but the underlying mechanisms have not been elucidated. In one study which showed that women perceive time as passing more slowly, the results may have been unfairly influenced as the women ran at a higher intensity than men (Hanson and Buckworth, 2016). There is a dearth of research examining the effect of the aforementioned factors on human time perception. Accurate perception of time is an important skill with many sports (i.e., time constraint regulations in basketball, North American football, tennis, gymnastics, figure skating, ice hockey, and others), and work environments (i.e., workers who need to synchronize their actions such as police and military). In addition, distortions of time perception may play a role in adherence to exercise. Individuals may be disinclined to continue with a healthy, exercise activity that seems to drag on. Thus, the objective of this review is to examine possible physiological and psychological factors affecting human perception of time and the mechanisms underlying time perception and distortion with activity (i.e., acute exercise and fatiguing exercise).

## Time Perception Models

### Scalar Expectancy Theory

**FIGURE 1:**
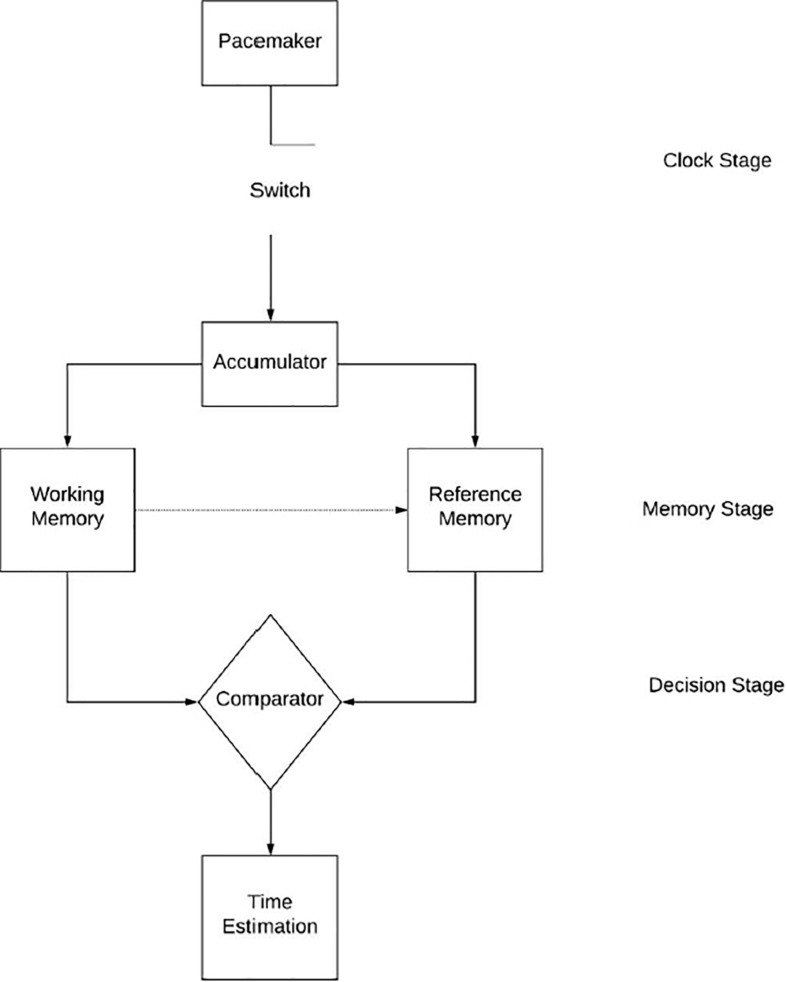
The processing of information inside of the hypothesized internal clock as it relates to time perception as described by the scalar expectancy theory.

The most referred model when discussing the effects of arousal on time perception is the scalar expectancy theory or pacemaker accumulator model (Grondin, 2010; Allman and Meck, 2012). The model divides the temporal processing system into three stages; clock, memory, and decision (Grondin, 2010), as illustrated in Figure 1. The clock stage begins at the onset of a signal whereby a switch, controlled by attention processes, closes and pacemaker pulses are collected into an accumulator (theoretical function of the brain presently without a specific location). If after some time, the signal acquires some added significance (i.e., feedback, changes in the environment) then the contents of the accumulator are transferred from working memory to reference memory for long-term storage (Allman and Meck, 2012); this entails the memory stage. The decision stage occurs when the duration is experienced again on a separate trial, a ratio-decision rule compares if the current contents of the accumulator fall below, meet or exceed a threshold of similarity (Grondin, 2010; Allman and Meck, 2012; Allman et al., 2014). This process creates our perception of time. The model attributes differences in time perception amongst individuals to differences in one’s attention, pacemaker speed, memory, and decision-making abilities (Allman and Meck, 2012).

The scalar expectancy theory is important in time distortion studies as it allows us to distinguish between the effects of attention and arousal on time perception (Zakay and Block, 1997). Attention is a function of the mode switch whereas arousal affects the speed of the pacemaker. Arousal increases the pacemaker speed, and thus the number of pulses collected in the accumulator (Gil and Droit-Volet, 2012; Lambourne, 2012). This arousal leads to a distortion in perceived time, specifically lengthening perceived time intervals (Gil and Droit-Volet, 2012). It is important to note that this effect has a multiplicative property (Zakay and Block, 1997); time distortion increases as the length of the stimulus duration increases. It can be hypothesized that exercise-induced arousal produces this effect, causing time distortion during exercise (Dormal et al., 2018).

### Striatal Beat Frequency Model

**FIGURE 2:**
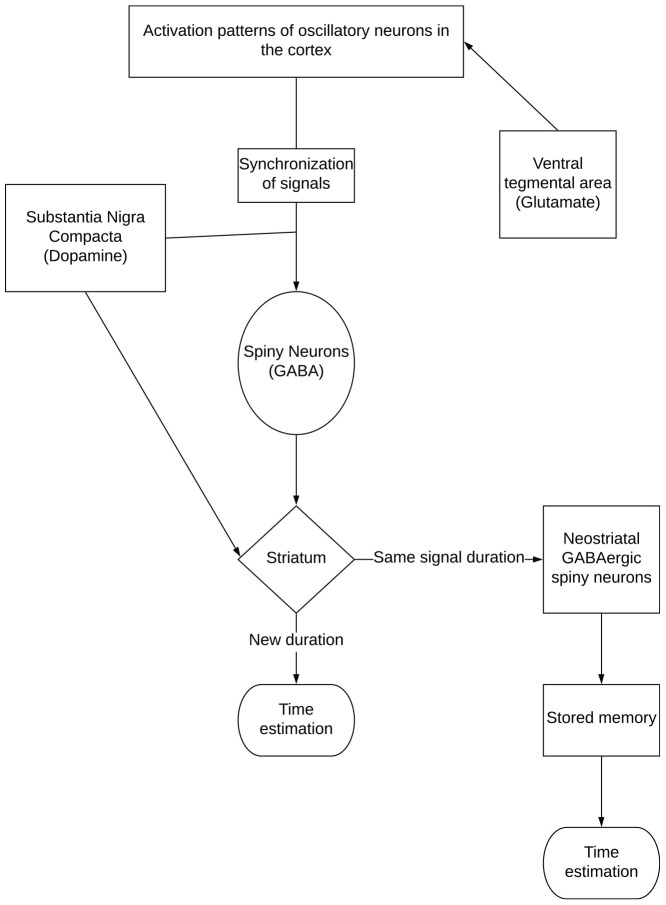
The neural regions associated with time perception and timing behavior as outlined by the striatal beat frequency model.

The striatal beat frequency model (SBF) embodies the interactive nature of timing networks. This model provides an advantage over the pacemaker accumulator model as it not only discusses timing behaviors, but specifies the neural regions involved in timing (Merchant et al., 2013) as outlined in Figure 2. The model says that clock speed is determined by levels of dopamine-glutamate activity in the substansia niagra compacta and ventral-tegmental area-cortical pathways. The process of timing begins with striatal spiny neurons that monitor activation patterns in the oscillatory neurons in the cortex, which are influenced by glutamate activity. When an interval begins, the oscillating neurons are synchronized, and the spiny neurons are reset by phasic dopaminergic input. When the target duration is reached, a pulse of dopamine is released; this strengthens the synapses in the striatum that are active (Meck, 2005). The rate of oscillatory activity is how time is perceived in the mind^6^. Similarly, mechanisms of long-term potentiation and depression are used to strengthen and weaken synaptic weights to produce a memory of the target duration (Matell and Meck, 2004). Later, when this same signal duration is timed again, neostriatal GABAergic spiny neurons compare the current activation pattern to the stored pattern to determine when the duration has been reached; when they match, spiny neurons fire to indicate the interval has elapsed (Matell and Meck, 2004; Meck, 2005; Merchant et al., 2013).

Distortion of time in this theory is attributed to context dependent activation dynamics, however, they are not yet fully understood (Merchant et al., 2013). It is believed that this time distortion effect may be caused by neural activity in different neural networks, which interfere with timing. This was observed in studies where emotionally charged stimuli distorted time perception due to activity in emotion and association networks (Dirnberger et al., 2012; Merchant et al., 2013). This model has not yet been used to describe exercise-induced arousal alterations of time perception, however, it has been used to describe differences in time perception between the sexes (Sandstrom, 2007; Pleil et al., 2011). Since the neural activity (i.e., motor unit recruitment, rate coding, intermuscular coordination, and others) associated with exercise differs dependent upon intensity, duration and type of activity (Behm and Sale, 1993), changes in time perception (distortion) might expected to be activity specific.

## Physiological Factors

### Aging

Adults often reminisce over how quickly time passes, and how time during their childhood seemed to be much slower in passing. This common complaint is so widely known that it seems to have become accepted in society as fact: as you age, time passes quicker. The effects of aging are easily observed with sensory changes, cognitive changes and loss of strength that varies in severity for the elderly (Jaul and Barron, 2017). These changes affect both physical and mental health, mobility, independence and overall quality of life.

There are clear age differences in time estimations of short intervals; older adults tend to estimate short intervals less accurately and with greater variability (Wittman and Lenhoff, 2005). These deficits are presumably a result from age-related changes in attention, working memory or the speed of information processing, as it relates to the pacemaker accumulator model of time perception. It must be noted that time perception for longer durations have not been studied with the elderly (Wittman and Lenhoff, 2005).

Interestingly, it seems that time perception may be related to age perception. In a study on elderly people (60–92 years) those who perceived themselves as younger than their chronological age perceived time as passing more quickly. In contrast, those who perceived themselves as being old, reported time to pass more slowly (Baum et al., 1984; Wittman and Lenhoff, 2005). In another study, elderly people between 60 and 84 years were asked to identify which period of time throughout their life seemed to pass the most slowly; childhood was the most popular answer (Tuckman, 1965). Two studies showed conflicting results when comparing young to elderly participants, where results indicated elderly perceived time to pass more quickly and that there were no differences in age (Tuckman, 1965; Tismer and Wahlen, 1979; Wittman and Lenhoff, 2005). The lack of consensus in the current research does not support the common view that time begins to pass more quickly as we age. However, these studies primarily focused on retrospective time estimation and not subjective time estimates (estimating time as is passes presently). Understanding the effects of subjective time perception, and potential distortion, in elderly persons may lead to a further understanding of neurological alterations as well as the sociological issues the aging population is currently facing and allow for more “elder-friendly” environments.

With the research conducted on aging and time perception, it is surprising that the interaction between age-related time perception distortions have yet to be studied during exercise or physical activity. This lack of research poses a significant problem for society today with the increasingly aging population. Many health issues related to aging are prevented or reduced in occurrence when individuals remain active and physically fit throughout their lives. The promotion of exercise programs for this population has become a priority in public health (Jaul and Barron, 2017).

If an individual’s time perception is altered unfavorably as they age, it may impact physical activity or exercise program adherence. For example, if elderly individuals cannot accurately perceive short intervals of time (Wittman and Lenhoff, 2005), similar to one set of an exercise, it may affect their ability to fully comply with exercise programs. This interaction has yet to be studied and so the severity of distortion of time perception for the elderly during physical activity/exercise is not yet known. Future research should seek to understand this interaction and generate suggestions as to how to ameliorate these issues that may be causing barriers to physical activity for the aging population. It would also be beneficial to investigate whether exercise could improve an elderly person’s perception of time during daily life.

### Youth

As mentioned previously, it is widely believed that time passes more slowly at younger ages. In a review by Droit-Volet (2013), an age-related increase in accuracy of time estimates and a reduction of temporal variability was found. A common study type that examines time perception in children is temporal reproduction tasks. During these reproduction tasks, children are asked to judge if the duration of a second stimulus is the same as an earlier stimulus. The data from these studies has been inconsistent in the determination of time estimate variability (over- or under estimations) for children (Crowder and Hohle, 1970; Droit-Volet, 1999, 2013; Chelonis et al., 2004). This inconsistency may be attributed to the studies containing participants of varying ages and to the progressive increase in the development of psychological functions between 3 and 10 years of age (Droit-Volet, 2013). Once again, there is a lack of information pertaining to how time is perceived for children during exercise.

### Sex

Sex differences in time perception during exercise have been explored in only one study. Hanson and Buckworth (2016) examined 11 men and 11 women recreational runners, who were instructed to run 75% of their average daily run distance at a self-selected pace (i.e., if their average daily run was 10 km, the participant ran 7.5 km). The participants were not informed of any time or distance intervals that passed and were only told when they had reached the distance endpoint. The results revealed that women ran at a higher self-selected pace than men, but also produced significantly lower time estimates. Hence, women perceived time to be passing by more slowly than their male counterparts. It must also be noted that these differences in time perception were present before, during, and after each run. These results may indicate that women focus more attention on time during exercise than men and thus theoretically accumulate pulses in the accumulator at a faster rate (scalar expectancy theory) (Hanson and Buckworth, 2016). One issue with this study remains that the men and women exercised at different intensities, as it was a self-selected intensity measure. Therefore, some of the difference in time perception may have been due to the fact that the women consistently selected higher intensities than their male counterparts.

Even so, on a physiological basis, men and women are quite different. Several studies on the influence of estradiol (estrogen: predominately female hormone), on time perception have been conducted in animal studies (i.e., rats) (Sandstrom, 2007; Pleil et al., 2011). These studies have collectively shown that estrogen can significantly slow the internal clock, meaning time is perceived as slower. This effect is explained through the striatal beat frequency model of time perception: estradiol interacts with dopamine activity, decreasing the internal clock speed (Pleil et al., 2011). Women’s higher levels of estrogen would differentially affect the structure and functioning of their striatal dopamine systems compared to men. Sandstrom (2007) describes this effect through the pacemaker model, detailing that the proportionality of the time shifts relative to the amount of estradiol administered suggests that the estradiol influences the speed of the internal pacemaker. Due to these factors, time perception may be fundamentally different between men and women. It has not yet been substantiated if the effect of estradiol is amplified in exercise-related time perception or if there is a constant difference between male and female time perception. Future studies may benefit from investigating this factor within female athletes to determine if natural estradiol levels affect time perception. Additionally, research on the administration of exogenous estradiol to male or transgender female athletes may allow for a better understanding of this phenomena, if it exists, during exercise. It is unknown if this difference in time perception affects the choice to exercise, stereotypical exercise activities of the sexes or affects exercise adherence differently between the sexes.

### Trained State

It may be assumed that highly trained persons have a more accurate perception of time during exercise (Edwards and McCormick, 2017). This effect may be due to task duration knowledge. This does not refer to the familiarization that occurs with task repetitions but the fact that one can extract specific knowledge about the duration of the task, after years of training (Tobin and Grondin, 2012). Elite athletes often receive repetitive feedback on time and duration, this feedback allows them to gain critical information about task duration that can later be used to estimate time more efficiently (Hicks and Miller, 1976; Edwards and McCormick, 2017). This effect was studied in a group of elite swimmers (Tobin and Grondin, 2012). These highly trained athletes were asked to identify their strongest and weakest stroke; with the idea that their strongest stroke would have been practiced more and thus the athlete would have more knowledge about the duration. The athletes were instructed to complete four separate 100 m trials: two with their best stroke, two with their weakest. Following the completion of each trial, athletes were asked to estimate the time it took them to complete the task. The results of this study showed a significant relation to time estimation and stroke, as the athlete’s best stroke was often estimated correctly or with very minimal error. The weaker stroke was estimated as less accurate. These results demonstrated that task duration knowledge improves performance in accuracy in time perception tasks. This may be explained through attentional effects, as the best stroke may have required less focus and attention due to more extensive training (Tobin and Grondin, 2012).

However, there has been no comparative investigations to examine if trained individuals perceive time differently than non-trained individuals during an unfamiliar exercise or activity. Other than task duration knowledge, there has been no investigation into time perception pertaining to trained state level. It is possible that well-trained individuals may respond differently as they are more accustomed to performing in an associative state (Edwards and McCormick, 2017). Results from such studies may help identify prescriptions of physical activity more suitable for a person’s trained state, as to maintain accurate time perception. Additionally, there is currently no evidence to suggest that a more accurate time perception during exercise improves performance (Tobin and Grondin, 2012).

### Body Temperature

Body temperature fluctuations occur normally throughout the day and in response to illness, such as fever, exercise, psychological stress, and external factors like environmental temperature (Nybo, 2012). Timing behavior can be manipulated through body temperature variations (Ghaderi, 2019), and there are several proposed explanations as to why this occurs.

It has been proposed that a temperature sensitive time mechanism exists within the brain (Tamm et al., 2015). A rationale originating from animal research suggests timing processes often are explained through scalar expectancy theory (Tamm et al., 2015). Time compression (perceived time is shorter than actual time) often occurs when core temperature is increased and is due to the pacemaker emitting pulses at a faster rate, similar to the fashion in which many physiological processes accelerate at higher temperatures (Tamm et al., 2014).

Classical physics can also be used to describe the mechanisms of this effect: an increase in enthalpy (temperature) leads to an increase in entropy, relating to the evidence that time passes faster with an increased entropy (Ghaderi, 2019). This is also consistent with the suggestion that time will travel in a certain direction when entropy is increased, as derived from the 2nd law of thermodynamics. Time is a psychologically interpreted process and so logically, the brain can be assumed to be the system. Animal models have shown that the cortex temperature can change daily by 0.5°C and it is estimated to be approximately the same for humans. These fluctuations can be achieved through cooling processes involving cerebrospinal fluid and heat exchange with the surroundings (scalp and skulls) and circulating blood and occur normally throughout the day in response to numerous physiological stimuli (Nybo, 2012). It is predicted that when the brain entropy and the environmental entropy are different, there is a mismatch between the two time-systems leading to differences in perceived and actual time (Ghaderi, 2019). However, in humans, brain temperature increases in parallel with body core temperature, which makes it difficult to examine the effects of cerebral temperature in isolation in many settings. Due to this phenomenon, an exercise-induced increase in core body temperature would create an increased brain temperature (hyperthermia) distorting time perception (Nybo, 2012). However, the effects of exercise-induced increases in cortical temperature have not been examined.

Circadian body temperature fluctuations have been shown to influence time estimation. An early study by Hoagland (1933), reported that overestimations of time intervals occurred in the afternoon, when the body temperature is naturally highest. Several studies have been conducted on body temperature and time by manipulating environment temperature, which in turn changes body temperature (Tamm et al., 2014, 2015). One study uncovered that heat acclimation may play a role in time distortion affects (Tamm et al., 2015). This study was conducted during the winter season and as such participants were no longer accustomed to extreme heat. Participants were instructed to complete a 30-min walk on a treadmill in a temperature-controlled room that was set to 42°C. The 10-day experiment allowed the participants to progressively acclimate to the hot and dry conditions. A significant main effect of core temperature was found on time, which concluded that the perception of time is susceptible to changes in prolonged exercise in the heat but these changes can be overcome as a result of heat acclimation. This is because the rate of the increase in core temperature was significantly slowed during exercise in hot conditions after heat acclimation (Tamm et al., 2015). However, there was no investigation on whether the body temperature increase caused by exercise was an additional factor or if it was simply the external heat that caused time perception issues. Results were also attributed to hormone responses under heat stress. It is important to note that this study showed that time distortions only occurred once a certain level of fatigue was reported, again indicating the important physiological role in time perception (Tamm et al., 2015). In another study by Tamm et al. (2014) using the same treadmill protocol, young men were asked to perform time reproduction tasks in varied temperature environments. The results of this study supported the same notion that temporal compression is related to a higher core temperature.

Less understood is the effects of exercise in colder environments on time perception. Research on time perception in cold environments has shown conflicting results as some studies have shown time deceleration while others has shown acceleration (Baddeley, 1966; Fox et al., 1967; Tamm et al., 2015).

Exercise produces a physiological arousal which, amongst many things, increases body temperature (Nybo, 2012). While exercise in hot environments has been extensively explored its effect on time perception in exercise is not well elucidated. It may be interesting to isolate this variable and determine whether core temperature changes resulting from exercise also change time perception. The aforementioned results support the importance of heat acclimation to ensure time acclimation. Further investigation into temperature-related time distortion may provide insight into how time is perceived over the course of a competition as body temperature increases or in shift sports, like ice hockey, where temperature fluctuations may occur. Additionally, this area of research should investigate the effect of warm-up procedures on time perception in competitions and practices. Alterations in time perception with exercise in cold temperatures should also be explored. The understanding of temperature effects has implications in extreme environments and situations where timing and accuracy is crucial: endurance, extreme sports, and military combat (Tamm et al., 2014, 2015).

### Exercise Intensity

The type and intensity of exercise performed has been shown to have an impact on time perception (Edwards and McCormick, 2017; Hanson and Lee, 2017; Karsilar et al., 2018). Although the research on this topic is limited, the inaugural study conducted by Edwards and McCormick (2017) demonstrated the effects of time perception distortion with varying exercise intensities. This study compared the Wingate anaerobic cycling test and an endurance exercise (rowing ergometry). Results of this study showed that with higher intensity exercise, time seemed to pass slower in relation to chronological time. This is seemingly a multiplicative effect (Edwards and Polman, 2013; Edwards and McCormick, 2017). This effect is attributed to the greater sensory awareness of physical discomfort during maximal or high intensity exercise (Edwards and Polman, 2013), due to the secretion of catecholamines causing a state of hyperarousal (Jansen et al., 1995). Hyperarousal causes a greater amount of neural information to be processed, which makes it feel as if more time has passed than in actuality [scalar expectancy theory: increased number of pulses collected in the accumulator (Gil and Droit-Volet, 2012; Lambourne, 2012)]. During high intensity/maximal exercise, this experience again is delivered into a shorter period, creating a heightened arousal and awareness, which leads to the aforementioned time distortion. It is important to note that this experiment was performed on recreationally active individuals, meaning the results of this study are only generalizable to this population (Edwards and McCormick, 2017). These results align with results from a study conducted by Hanson and Lee (2017), where time seemed to pass by more slowly as a higher intensity was completed and a higher RPE was reported. Another study that examined walking speed as a measure of intensity demonstrated that with an increase in walking speed, time subjectively dilated (passed more slowly) (Karsilar et al., 2018). Interestingly, the effect of exercise intensity on time perception was most prominent in long duration, endurance-type exercises (e.g., rowing ergometer tests) (Edwards and McCormick, 2017). The mechanisms underlying this discovery have not yet been substantiated. The discovery of these effects may have implications in accurate pacing in races, further demonstrating the importance of external feedback of chronological time so that the athlete may accurately judge the duration of the event, evaluate resources and produce their desired outcome (Edwards and McCormick, 2017).

### Contraction Types and Movements

Different types of contractions and movements (i.e., isometric, dynamic, cyclical, and rhythmic) can differentially affect fatigue and force production (Halperin et al., 2015). However, within the existing research there has yet to be a comparative study between contraction types or movements and time perception. Two studies investigated the effects of tapping, vibration and static contractions on time perception. Tomassini et al. (2014) investigated how the motor system encodes and influences sense of time by utilizing tactile stimulation. In the movement condition, two pairs of tactile taps were presented randomly to the right or left index finger while the isometric contraction condition tactile stimuli was delivered to the right index finger. Their results demonstrated that perceived duration decreased during an isometric force production task but not in the control setting. However, these results of temporal distortion were alluded to be caused by the tactile stimulation (Tomassini et al., 2014; Merchant and Yarrow, 2016). Another study investigated similar concepts by contrasting a static condition versus a vibrotactile stimulus on a finger (Press et al., 2014). Results showed that the application of the vibrotactile stimulus increased the perceived duration. This demonstrates the effects of tactile stimulation while contracting (isometrically) on time perception (Press et al., 2014; Tomassini et al., 2014; Merchant and Yarrow, 2016).

These studies did not directly investigate the effects of contraction type nor did they compare different contraction types. The added variable of tactile stimulation does not allow for independent analysis comparing the effect of time perception during isometric and dynamic conditions. As such, there is currently no information regarding the effects of differing contraction types and movements on time perception. Future research should look to uncover these potential effects as it may be beneficial for the ordering of exercise programs.

### Psychological and Emotional Influences

The old saying, ‘time flies when you’re having fun’ may have some credence, as time perception accelerates (flies) when you are experiencing goal motivated fun (Gable and Poole, 2012). With a positive emotional state, the experience of time is seemingly accelerated. This may be attributed to attentional aspects, which interfere with the process of the pacemaker accumulator model (Hanson and Lee, 2017). A goal-oriented activity may cause one to be distracted, which means the pulses may start being collected at a later point in time that does not accurately represent the start of the interval (Gable and Poole, 2012; Hanson and Lee, 2017). While this hypothesis has not been tested in relation to exercise or exercise-related time perception, it may be an interesting area for future research to investigate. Many individuals are intrinsically motivated and enjoy the challenging nature of exercise. These individuals may perceive time as passing more quickly than those who just exercise for other reasons, however, no studies have examined this hypothesis yet.

When people are experiencing a negative emotional state, like fatigue, fear or boredom, they feel as if time is passing by more slowly than in reality (Kent et al., 2019). Again, attentional aspects of timing behavior may dictate that this is caused by an increased attention to time (Gable and Poole, 2012; Droit-Volet and Berthon, 2017; Hanson and Lee, 2017) which causes the accumulation of pulses to occur earlier than if one was distracted by a goal-oriented activity. Emotional arousal has been demonstrated to distort time perception (Droit-Volet and Berthon, 2017).

Fear is considered a negative emption (Hanson and Lee, 2017; Kent et al., 2019), and it has been shown to speed up the internal clock processing and create the perception of a longer duration (Fayolle et al., 2015). This can be explained through the scalar expectancy theory, where more pulses would be accumulated at a faster speed due to the emotional arousal and thus increase the length of perceived time. In a study conducted by Fayolle et al. (2015), individuals were asked to judge the length of an interval, during which an electric shock would be administered. Results showed that those trials with an electric shock were judged to be longer than those without; this is attributed to emotion-related arousal. These findings are consistent with the Droit-Volet and Berthon (2017) proposition that emotional arousal affects time perception. It is important to note that these effects of emotional arousal, from fear or otherwise, have not been studied in exercise conditions. Future studies should seek to understand if these emotional arousal-related time distortion effects are amplified by the physiological arousal created by exercise. The implications of this research may prove important in both rehabilitation, clinical and fitness settings. A person who is beginning physical therapy, practicing their prescribed exercises at home or new to joining a fitness facility, may experience some level of fear or anxiety. If the effect of time perception lengthening is present in these situations, especially during the actual exercise, it may be discouraging to the beginning exerciser.

Additionally, several studies have investigated lack of arousal or boredom (Danckert and Allman, 2005; Zakay, 2014; Thönes and Oberfeld, 2015; Hanson and Lee, 2017; Kent et al., 2019). Boredom is defined as a period of time when information processing loads are low (Zakay, 2014). Danckert and Allman (2005) demonstrated that boredom is relevant in time perception for intervals between 15 and 60 s, in which time feels as if it is flowing more slowly as more attention is given to time. These results were supported by several studies (Zakay, 2014; Thönes and Oberfeld, 2015; Kent et al., 2019). There are currently no studies examining the effects of tedium or boredom on time perception during exercise. Results of studies investigating this may prove useful in creating well-structured exercise programs that work to maintain an accurate time perception.

Indeed, individuals who are bored and persons with depression exhibit similar time perception distortions studies (Danckert and Allman, 2005; Zakay, 2014; Thönes and Oberfeld, 2015; Kent et al., 2019) and the two are often studied together. Depression is also known to decelerate time, however, it is hypothesized that this effect is amplified by intrusive recall of negative memories (Kent et al., 2019). It may be interesting to investigate if exercise is beneficial for persons with depression’s time perception and timing behaviors. Research should seek to uncover the most beneficial ways to exercise (type and intensity) so that time distortion is minimized for persons with mental or emotional deficits.

### Perceived Fatigue

Mentally fatiguing tasks or cognitively demanding tasks are known to hinder performance in physical tasks when performed concurrently or continuously (Halperin et al., 2015). Physical tasks also create cognitive strain due to the continuous attention required to maintain force production and proper form and the act of inhibiting the urge to quit when exercise becomes strenuous and uncomfortable. These cognitive demands from physical exertion create mental fatigue which may increase one’s perceived rate of exertion and impair mental processes, like timing behavior. This feeling of fatigue also initiates an increase in stress-induced arousal level (Tamm et al., 2014, 2015). Stress-induced arousal has been hypothesized to increase the speed of the internal clock by increasing the number of pulses emitted and then accumulated. This ideology is seen within an experiment conducted by Tamm et al. (2014) which detailed the interaction between perceived fatigue and exercise-related time perception distortion. Results of this study showed that temporal compression occurred in a hot environment but only once a certain level of fatigue was perceived by the athletes, recorded with rate of perceived exertion (RPE) scale. The level of perceived fatigue needed for temporal compression to occur was not reported in the study. This is currently the only study that examines this concept and as such, its results must be interpreted with caution as there were multiple variables present (heat, interval reproduction). Further research is needed to verify these findings.

### Attentional Effects

As stated in the scalar expectancy theory model, attention plays an important role in time perception. When more attention is given to time, it seemingly slows time perception. In contrast, when less attention is given to time, it seems to speed up the perception of time (Hanson and Lee, 2017). This attentional effect has been attributed to the mechanisms underlying many of the time perception distortion effects seen with age, sex, temperature, psychological, and trained state (Danckert and Allman, 2005; Wittman and Lenhoff, 2005; Gable and Poole, 2012; Tobin and Grondin, 2012; Zakay, 2014; Fayolle et al., 2015; Thönes and Oberfeld, 2015; Hanson and Buckworth, 2016; Droit-Volet and Berthon, 2017; Hanson and Lee, 2017; Kent et al., 2019).

One study specifically investigated the attentional aspect during exercise by utilizing the distraction of music (De Bourdeaudhuij et al., 2002). This study looked at the effect of distraction on treadmill running time in obese children and adolescents. Participants were instructed to complete a treadmill test at the beginning and end of a residential treatment plan. This test comprised an incremental protocol, which was set to each individuals’ pre-determined running time till exhaustion. Two separate trials were conducted; the control with no distractions and the experimental trial where participants selected music to play while they ran. Results showed that the participants were able to run much longer in the distracted setting (with music). These findings align with previous studies that showed improved running performance in distracted settings (Masters and Ogles, 1998; Maddigan et al., 2019). This effect was attributed to the lengthened amount of time it takes to perceive bodily symptoms and sensations while distracted (De Bourdeaudhuij et al., 2002), but the study did not comment on the potential change in time perception that the individuals may have experienced. However, this effect (lengthening the time it takes to perceive bodily symptoms and sensations) has been noted in the past to affect time perception by slowing down the pacemaker, leading to the sense that less time has past (Edwards and McCormick, 2017).

While this study did not investigate time perception differences between the trials, it may be assumed to be similar to other studies with attentional deficits (Danckert and Allman, 2005; Wittman and Lenhoff, 2005; Gable and Poole, 2012; Tobin and Grondin, 2012; Zakay, 2014; Fayolle et al., 2015; Thönes and Oberfeld, 2015; Hanson and Buckworth, 2016; Droit-Volet and Berthon, 2017; Hanson and Lee, 2017; Kent et al., 2019); time perception may have also been altered. Future research should look to explicitly investigate the effects of attention and time perception during exercise. Applications of this research may be important in rehabilitation and fitness facility settings and provide insight on how to ameliorate their environments for optimal time perception. Research should also seek to discover whether an attention-based time perception distortion, such as music, is beneficial for exercising or if it may hinder aspects of performance improvement.

## Conclusion

The perception of time can impact success in sport, fitness and work. Two predominant theories (scalar expectancy theory and striatal beat frequency model) emphasize the perception of the number of events in a period and the role of neurotransmitters in activating and coordinating cortical structures, respectively. The extent of arousal can alter time perception and both physiological and psychological arousal are evident with work and exercise. Time perception with work and exercise can integrate a variety of factors including age, sex, body temperature, state of health and fitness, mental concentration, and exercise intensity level. Further work is needed on these factors individually as well as their roles with exercise; an area that has received scant attention in the literature.

## Author Contributions

All authors listed have made a substantial, direct and intellectual contribution to the work, and approved it for publication.

## Conflict of Interest

The authors declare that the research was conducted in the absence of any commercial or financial relationships that could be construed as a potential conflict of interest.
